# Supplementary figures and images for: Evolution-guided mutagenesis of the cytoplasmic incompatibility proteins: Identifying CifA’s complex functional repertoire and new essential regions in CifB
Source: PLoS Pathog. 2020 Aug 19;16(8):e1008794. doi: 10.1371/journal.ppat.1008794 (PMC7458348; doi:10.1371/journal.ppat.1008794)

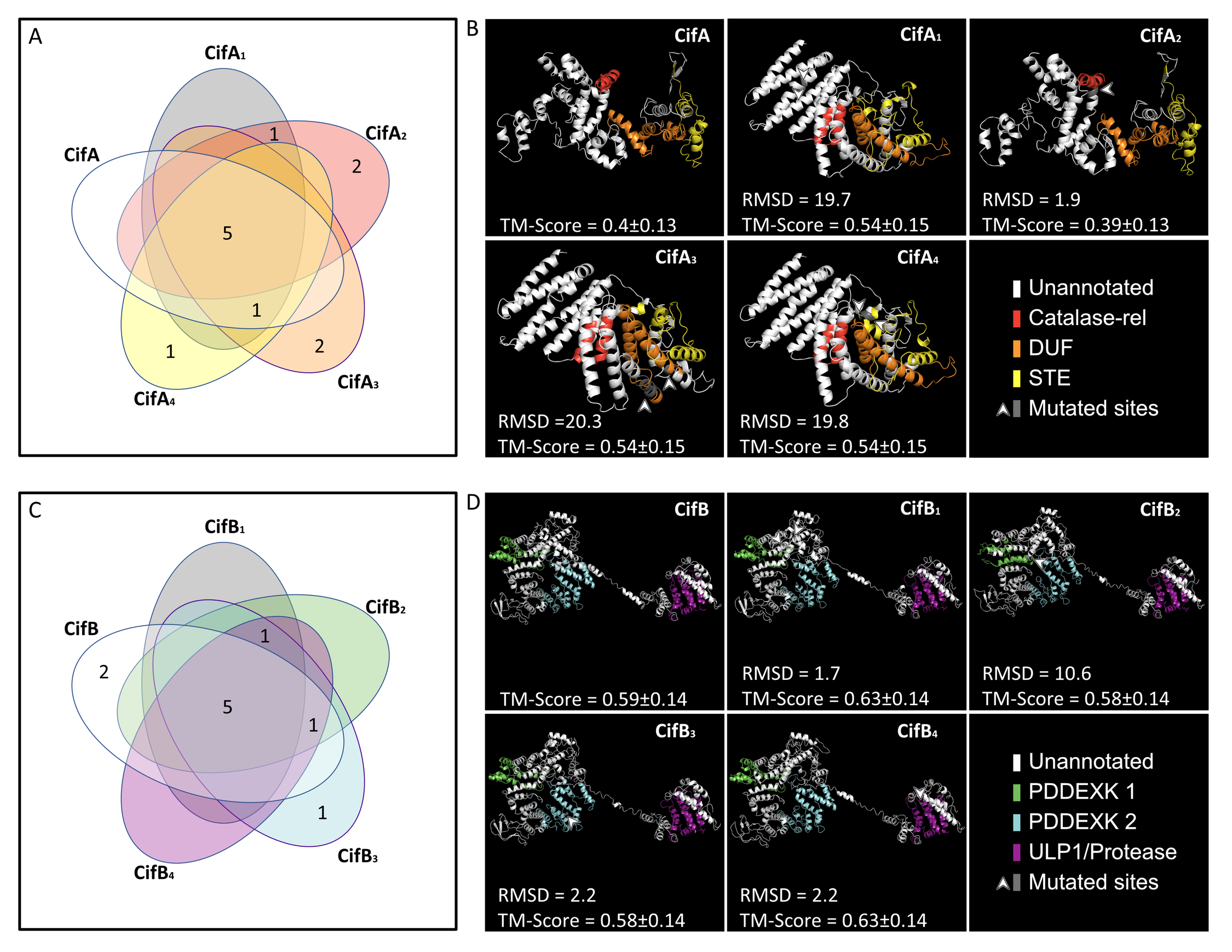

Supplement: S1 Fig — (A, C) Venn-diagrams showing the number of PDB hits shared between wild-type and mutant (A) CifA and (C) CifB proteins. (B, D) I-TASSER used these PDB hits to generate structural predictions for (C) CifA and (D) CifB. TM-scores range from 0–1 where 1 is the highest confidence. RMSD scores are from pairwise alignments of mutant proteins with the wild-type in PyMol. Higher RMSD scores represent more distance between the superimposed proteins. Mutated sites in the tertiary structure are indicated with a white arrow. Domain annotations were based on previous sequence analyses [27]. Details regarding the PDB hits are reported in S2 Table. (TIF) [file ppat.1008794.s001.tif]
