# Supplementary material for: Evolution-guided mutagenesis of the cytoplasmic incompatibility proteins: Identifying CifA’s complex functional repertoire and new essential regions in CifB
Source: PLoS Pathog. 2020 Aug 19;16(8):e1008794. doi: 10.1371/journal.ppat.1008794 (PMC7458348; doi:10.1371/journal.ppat.1008794)
Supplement: Attachment — Submitted filename: 2020_PLOS_Pathogens_Mutant_Response_Final.pdf [file ppat.1008794.s007.pdf]

VANDERBILT UNIVERSITY

Seth R. Bordenstein, Ph.D.
Centennial Professor of Biological Sciences
Professor of Pathology, Microbiology, and Immunology
Director, Vanderbilt Microbiome Initiative
Associate Director, Vanderbilt Institute for Infection, Immunology and Inflammation

June 29, 2020

Dear Colleagues and Editor of *PLOS Pathogens*:

Thank you very much for handling the review of our manuscript during this challenging time. We were asked to make several minor changes to the manuscript. We thank all of the reviewers for their thoroughness and critique, and we believe these comments have improved the work.

Reviewer #1:

This paper elegantly uncovers and beautifully explores the functional domains of the two phage-derived, Wolbachia-encoded proteins CifA and CifB that act as the two key effector proteins for directing cytoplasmic incompatibilities in insects.

Based on their earlier and other´s prior published data they systematically analyzed the protein sequences of CifA and CifB for highly conserved sites across phylogenetically distant Cif genes, site-mutagenized such candidate sites in 4 domains of each wMel gene and expressed them under the control of nanos in transgenic Wolbachia-uninfected flies. By this systematic in vivo approach, the authors have uncovered novel and important aa domains in these phage-derived proteins that affect either the CI, the Rescue or both Wolbachia-expressed phenotypes. Thereby they could demonstrate that in contrast to earlier expectations also the CifA protein, which has been regarded as a pure antitoxin in the TA model, has at least three functional domains that affect CI and two of them also the rescue phenotype. By this, these data further support their "Two-by-One" genetic model of CI.
In addition, they elegantly showed that beside the earlier characterized Ubiquitin-like-specific protease domain earlier proposed by Beckmann as the "enzymatic warhead" for CI three other domains are functionally crucial for CI in vivo.

To sum up, these novel and exciting data are important and pivotal for deepening our understanding in CI and hence this study sets the corner stone for further studies in deciphering the exact biochemical and cell biological mechanisms of these two Type 1 master genes that are driving reproductive parasitism in insects.

We thank the reviewer for their kind words, much appreciated.

Reviewer #1: Minor Issues: Editorial and Data Presentation Modifications
Line 272: since this part covers CifA and CifB proteins change Fig S1 A to Fig S1A,C and in line 274 Fig S1B to FigS1B,D.

Thank you for noting this error. It has been corrected as suggested.

Line 324ff: For a broader audience of readers it would be helpful to introduce the different mechanistic models for CI already in their introduction and phrase out the abbreviations used (i.e., HM and TA).

Thank you for the recommendation. We replaced HM with Host-Modification and TA with Toxin-Antidote throughout the manuscript. We have also added a paragraph on the HM and TA models to the introduction, as requested.

Reviewer #2:

The manuscript by Shropshire et al. investigates the phenotypic impact of evolution-guided introduced mutations in the two CI associated genes of Wolbachia wMel, CifA and CifB, when transgenically expressed in Drosophila melanogaster. This is the first study that investigates the effect on CI of replacements of conserved amino acid residues across several domains/regions of the CifA and CifB proteins. The results suggest that both proteins are needed for the induction of CI, and that residues along the whole CifB protein, and a large part of CifA are necessary for CI induction, whereas only the N-terminal part of CifA is involved in rescue of CI.

The results support the Two-By-One model, that suggest that both proteins are necessary for CI induction, whereas CifA is responsible for rescue. Additionally, it suggests that several regions/residues of each protein, with different predicted functions or no predicted function at all, has a large impact on the phenotypic expression of CI. Finally, it implies that there are sites in CifA that are only involved in CI induction.

Overall, I think the authors provide good arguments for their conclusions, which are clearly supported by their results.

Given the potential utility of the CI mechanism as a biological control method for insect pests and disease vectors, studies that contribute to further understanding of the mechanism and functions of the Cif proteins is of high significance.

We thank the reviewer for their kind words, much appreciated.

Reviewer #2: Minor Issues: Editorial and Data Presentation Modifications

• L362: From looking at figure 2, 34,05% survival is wrong for cifA2 rescue. It looks more like 28% or so to me. Additionally, I think it is a bit confusing that the authors report the percent of embryos that die in the previous sentence (also 34,05%) and the percent that survive in this one. I suggest that the authors use the same measure (i.e. hatch rate as in figure 2), when referring to both mod and resc functions.

Thank you for noting this error. We edited this with the correct value. We also opted to use percentage of embryos hatched in both cases for consistency, as suggested.

• The methods need to be more clearly described overall. Even though many of the methods have been used in other previous publications, I believe that it should be possible to get the main points without reading several additional publications.

Thank you for the recommendation. We expanded the methods.

o It is possible to infer from the results section which crosses were made, but it is not really described in the Methods section.

Thank you for the note. We recognize that this is the case and struggled initially with the balance of detail in this section. Adding the details of every cross would make the methods complicated and difficult to read. That being said, the specific details of the crossing are available in the supplementary data provided with this manuscript (Supplementary Data File 1). We also added a note in the methods that the genotypes associated with each crossing scheme are available in the supplementary data file.

o How was the expression of the transgenic genes tested?

Thank you for the question. Prior to experimentation, all gene constructs were Sanger sequenced and confirmed to contain the expected sequence. We previously confirmed expression in subsets of these lines in Shropshire et al. 2018, PNAS and Shropshire and Bordenstein 2019, PLOS Genetics. Since the same driver line is used across all crossing types, and the same plasmid constructs and insertion sites were used in each transgenic line, we used phenotypic expression of CI and rescue in our control lines to confirm expected expression since our design achieves high efficiency in CI and rescue levels.

o The source data for Fig2 and Fig3 contains repeats, but I can't find that this is described anywhere in the text.

Thank you for the comment. This was noted in the last line of each figure legend. Figure 2B was repeated three times, Fig 2C was repeated twice, and Fig. 3D was repeated three times.

• I am wondering why the authors chose to do the crosses with the cifA:B dual expression transgenic flies and CifA-types rather than with Wolbachia-infected male when testing the rescue phenotype. Wouldn't it be relevant to test the CifA variants against the natural Wolbachia infection? Or at least as well as the transgenes, since the expression from Wolbachia might be different than the transgenic expression of CifA and CifB in several ways. I suggest that the authors add at least something small in the discussion about this.

This is a great question. We reasoned that it would be preferable to cross with flies inducing transgenic CI or rescue for two reasons. First, in our view, transgene-induced phenotypes provide a more reductionist comparison since we are comparing transgenic mutants to transgenic wildtypes without the confounding impacts of *Wolbachia* infection in the system. Simply put, this is an issue of comparing apples to apples. Based on our prior work, transgenic expression is sufficient to induce CI and rescue. Second, transgenic CI and rescue is consistently very strong relative to *w*Mel infection that can vary if not well controlled. Thus, it would be

difficult to interpret weaker reductions in *w*Mel phenotypes relative to transgenic ones. We have added text to the results section associated with Fig. 2C that explains this reasoning.

• The illustration in Figure 1B is the same as used in another publication Shropshire and Bordenstein, PLoS Genetics 2019, which could be noted in the legend.

Thank you for this recommendation. We have cited this paper in the legend.

Yours sincerely,

Seth R. Bordenstein
Email: s.bordenstein@vanderbilt.edu
Phone: 615.322.9087

Dylan Shropshire
Email: dylan.shropshire@vanderbilt.edu
Phone: 615.322.9094
